# Convalescent Adaptive Immunity is Highly Heterogenous after SARS-CoV-2 Infection

**DOI:** 10.21203/rs.3.rs-3222112/v1

**Published:** 2023-08-24

**Authors:** Paige K. Marty, Balaji Pathakumari, Maleeha Shah, Virginia P. Keulen, Courtney L. Erskine, Matthew S. Block, Pedro Arias-Sanchez, Patricio Escalante, Tobias Peikert

**Affiliations:** Mayo Clinic; Mayo Clinic; Mayo Clinic; Mayo Clinic; Mayo Clinic; Mayo Clinic; Mayo Clinic; Mayo Clinic; Mayo Clinic

## Abstract

Optimal detection strategies for effective convalescent immunity after SARS-CoV-2 infection and vaccination remain unclear. The objective of this study was to characterize convalescent immunity targeting the SARS-CoV-2 spike protein using a multiparametric approach. At the beginning of the pandemic, between April 23, 2020, to May 11, 2020, we recruited 30 COVID-19 unvaccinated convalescent donors and 7 unexposed asymptomatic donors. Peripheral blood mononuclear cells (PBMCs) were obtained from leukapheresis cones. The humoral immune response was assessed by measuring serum anti-SARS-CoV-2 spike S1 subunit IgG semiquantitative ELISA and T cell immunity against S1 and S2 subunits were studied by IFN-γ Enzyme-Linked Immune absorbent Spot (ELISpot), flow cytometric (FC) activation-induced marker (AIM) assays and the assessment of cytotoxic CD8^+^ T-cell function (in the subset of HLA-A2 positive patients). No single immunoassay was sufficient in identifying anti-spike convalescent immunity among all patients. There was no consistent correlation between adaptive humoral and cellular anti-spike responses. Our data indicate that the magnitude of anti-spike convalescent humoral and cellular immunity is highly heterogeneous and highlights the need for using multiple assays to comprehensively measure SARS-CoV-2 convalescent immunity. These observations might have implications for COVID-19 surveillance, and optimal vaccination strategies for emerging variants. Further studies are needed to determine the optimal assessment of adaptive humoral and cellular immunity following SARSCoV-2 infection, especially in the context of emerging variants and unclear vaccination schedules.

## Introduction

Effective antigen-specific adaptive immunity is essential for the successful clearance of severe acute respiratory syndrome coronavirus 2 (SARS-CoV-2) infection. Convalescent and vaccine-induced adaptive immune responses are typically characterized by both humoral and cellular immunity.^1^ CD4^+^ and CD8^+^ T-lymphocytes represent key components of the cellular anti-SARS-CoV-2 immune response. Through cytokine production and cytotoxicity, these cells limit disease progression, promote viral clearance and contribute to the development of SARS-CoV-2-specific immune memory.^2,3^ While many studies have investigated adaptive immune responses following SARS-CoV-2 infection, comprehensive comparative immune profiling data of unvaccinated convalescent COVID-19 patients characterizing individual adaptive convalescent immune responses remains sparse. Several studies have utilized IFN-γ enzyme-linked immunosorbent spot (ELISpot), intracellular staining of cytokines, or non-cytokine activation-induced marker (AIM) by flow cytometry (FC) on peripheral blood mononuclear cells (PBMCs) to characterize anti-SARS-CoV-2 T-lymphocyte responses.^4–7^ Following SARS-CoV-2 infection, these immune profiling methods demonstrated variable cellular adaptive immune responses among patients and vaccine recipients, and unfortunately, no clear universal correlate of protective immunity has been validated and standardized.^8^

Lymphopenia and immune dysregulation have been widely reported as features of acute and subacute COVID-19.^9,10^ Furthermore, cellular immune responses also vary based on the timing of the infection and disease severity as well as other individual host factors.^7,11^ While the numbers of circulating CD4^+^ and CD8^+^ T lymphocytes are frequently reduced in patients during the acute and subacute phases of moderate or severe SARS-CoV-2 infections,^5,12^ robust and diverse antibody and T cell responses targeting multiple structural and non-structural regions of SARS-CoV-2 are present in a majority of convalescent COVID-19 patients, regardless of disease severity.^13–15^ While convalescent cellular immunity includes diverse CD4^+^ and CD8^+^ T-cell epitopes, these responses may diminish over time.^6,16^

Adaptive antibody responses have also been widely studied in response to SARS-CoV-2 infection and vaccination. Specifically, the production of anti-spike protein IgG, blocking the entry of the SARS-CoV-2 virus into the host cell, has been investigated extensively.^17,18^ However, antibody titers and persistence of humoral immunity longevity have been variable, and post-infection and post-vaccination antibody levels are transient leading to re-infection, especially with emerging SARS-CoV-2 variants, including the Omicron subvariants.^19–29^ Similar to antigen-specific T-cell responses, neutralizing anti-Spike antibody titers have also not consistently been associated with disease severity, although patients with persistently elevated anti-spike IgG levels may be protected from reinfection following asymptomatic-to-moderate COVID-19.^7,22,23^ Furthermore, while some studies suggested that anti-Spike neutralizing antibody titers correlate with SARS-CoV-2 antigen-specific T-cell responses, others have failed to observe this association.^30^ Other immunogenic structural proteins of the SARS-CoV-2 virus, including membrane proteins, can elicit B-cell and CD4^+^ and CD8^+^ T-cell responses, but some of these immune responses may represent cross-reactive T-cells induced by epitopes from structural proteins of other coronaviruses.^6,20,31–33^ In this context, a longitudinal study that included patients recently infected demonstrated that very early induction of functional SARS-CoV-2 specific cellular response detected by IFN-γ ELISpot in newly diagnosed COVID-19 patients was associated with rapid viral clearance and a milder disease course. ^34^

While most previous studies have utilized one or two immune profiling techniques to measure convalescent antigen-specific immunity after COVID-19 infection and vaccination, data providing a more comprehensive characterization of SARS-CoV-2 antigen specific cellular immune responses in unvaccinated convalescent patients is sparse.^4,6,7,13,17,22,35,36^ At this stage of the pandemic, unless stored samples from previously unvaccinated convalescent donors collected early during the pandemic are available, the high prevalence of COVID-19 vaccination and re-infection with newly emerging SARS-CoV-2 variants will confound the characterization of convalescent immunity in response to COVID-19.

Herein we present the individualized comparison of comprehensively characterized anti-Spike SARS-CoV-2 antigen specific cellular immune responses among convalescent patients who successfully recovered from COVID-19 early during the pandemic (April-May 2020). This immunoprofiling comparison also includes the measurement of T-cell cytotoxicity among the subset of HLA-A2 positive patients.

## Materials and Methods

### Participants

Peripheral blood mononuclear cells (PBMCs) were obtained from leukapheresis cones of 30 COVID-19 convalescent donors who were enrolled in the Mayo Clinic COVID-19 convalescent plasma program between April 23, 2020 to May 11, 2020 and from 7 COVID-19 unexposed Mayo Clinic Blood Bank platelet donors. The study was reviewed by our Institutional Review Board and due to the de-identified nature of the samples, and procedural waste (leukapheresis cones of convalescent plasma and platelet donors), the study was not considered to represent human research. However, all study subjects provided written informed consent to donate either platelets (unexposed individuals) or plasma (convalescent patients) in the blood bank as part of the Mayo Clinic Blood Bank or the Mayo Clinic COVID-19 convalescent plasma donor program, respectively. All the methods were carried out in accordance with relevant guidelines and regulations after obtaining approval and recommendations from Institutional Review Board of Mayo Clinic. Consequently, other than age and gender, information regarding the clinical presentation, disease severity of COVID-19 infection or comorbidities was not available. All convalescent donors had a documented history of SARS-CoV-2 infection with positive nasopharyngeal swab PCR testing followed by a full clinical recovery. This was defined by a minimum of 28 days after complete resolution of symptoms, or negative SARS-CoV-2 nasopharyngeal swab PCR testing twice and a minimum of 14 days prior to plasma donation and PBMC collection. The first confirmed COVID-19 case was reported on March 5, 2020 in the state of Minnesota (www.health.state.mn.us/diseases/coronavirus/situation.html#cases1), and thus, these 30 donor samples were most likely collected from COVID-19 early convalescent subjects at the beginning of the pandemic in our region. Control blood donors were recruited to donate platelets as part of the Mayo Blood Bank. The samples were collected prior to the beginning of the pandemic and so, by definition, they were most likely unexposed to SARS-CoV-2.

### PBMC preparation from cones

Blood cells were obtained from Trima cones, diluted in PBS and isolated using density centrifugation over Ficoll-Hypaque (Sigma-Aldrich, Saint Louis, MO) at 450g for 30 mins. The buffy coat was collected, washed twice in PBS and viability was checked with trypan blue. Fifteen million cells per vial were frozen in 1 mL of Cosmic Calf serum (Thermo Fisher Scientific, Waltham, MA) containing 5% DMSO using a Mr. Frosty (Thermo Fisher Scientific, Waltham, MA) freezing container overnight at −80°C. The following day, cells were stored in liquid nitrogen until use.

### Anti-SARS-CoV-2 spike antibody measurement

Serum anti-SARS-CoV-2 spike S1 subunit IgG semiquantitative ELISA was conducted according to the manufacturer’s instructions (Euroimmun, Lubeck, Germany). Testing results were only available for COVID-19 convalescent patients and results are given as the ratio of patient sample/control sample.

### Identification of HLA-A2-positive patients and HLA-A2-binding peptides

One million PBMCs from each sample were washed in staining buffer (PBS + 1% BSA) and incubated for 30 mins with PE Mouse anti-Human HLA-A2 antibody (BD Pharmingen, San Diego, CA). After incubation, cells were washed in staining buffer and fixed with 0.5% paraformaldehyde. The samples were run by the Mayo Clinic Microscopy and Cell Analysis Core on a FACSCanto machine. Files were then analyzed using FlowJo^®^ (Tree Star, Ashland, OR). The amino acid sequence for SARS-CoV-2 spike protein was input into the NetMHC-4.0 algorithm as previously described.^37^ Peptides of 8–11 amino acids with a predicted affinity to HLA-A2 of < 40 nM were identified (Table 1). Peptides were synthesized by the Mayo Clinic Proteomics Core.

The PBMCs were stimulated with multiple antigens and controls for a total of 40 hrs. The antigens used included: 1) SARS-CoV-2 Spike protein S1 subunit (MyBiosource.com: Gln14-Arg685, recombinant protein #MBS8574750, 0.1mcg/ml); 2) SARS-CoV-2 Spike protein S2 subunit (MyBiosource.com: S2,685–1211aa, recombinant protein #MBS569936, 0.1mcg/ml); 3) SARS-CoV-2 Spike peptides (Table 1, **10**mcg/ml, used in HLA-A2-positive patients only); 4) tetanus toxoid (TT, Biological Labs, #191A, 0.1mcg/ml); and 5) media (unstimulated control).

### FC AIM Assays

Antigen-stimulated PBMCs were analyzed by FC with AIM assays to identify the following subsets among CD4^+^ and CD8^+^ T cells upregulating the following surface markers: CD25^+^CD134^+^, CD25^+^ PD-L1^+^, and CD11a^+^PDL1^+^. Upon staining, cells were fixed with 0.5% paraformaldehyde and acquired at least 250,000 cells by BD LSRFortessa (BD Bioscience, San Diego, CA, USA). The FCS files was analyzed using FlowJo^®^ (Tree Star, Ashland, OR) and Kaluza^®^ analysis software (Beckman-Coulter, Inc, Brea, CA). The net percentage of antigen specific surface markers was calculated by subtracting the unstimulated (nil) from stimulated values. Receiver operating characteristics (ROC) curve analysis defined the cut-offs of each antigen condition to best differentiate study groups of interest as previously described.^38^ Individual positive AIM assay results were determined by comparing the individual values of the AIM assays minus nil with the best cut-offs for the corresponding CD4^+^ and CD8^+^ subsets and antigen stimulation condition. Minimal detection thresholds were determined as described by Bowyer *et al*. ^39^

#### IFN-γ ELISpot assay

In 96-well plates, 2.5 × 10^5^ cells per well of antigen-stimulated and control PBMC samples were added in 200 μL media and incubated at 37°C for 24 hrs. Each sample was done in triplicate. ELISpot plates (Millipore, Billerica, MA) were coated with 10 μg/mL IFN-γ capture antibody (MabTech, Mariemont, OH, USA) and incubated overnight. After 24 hrs, the ELISpot plates were washed with PBS and blocked with culture medium containing 10% FBS for 2 hrs. Activated PBMC samples were transferred to the ELISpot plate and incubated for 24 hrs at 37 °C, 5%CO2. Following incubation, the plates were washed with PBS containing 0.05% tween-20 and 2 μg/mL of biotinylated secondary antibody for IFN-γ (MabTech, Mariemont, OH, USA) was added. The plates were incubated for 2 hrs at 37 °C followed by another wash. Next, 1 μL of Streptavidin-horseradish peroxidase (BD Pharmingen, San Diego, CA) per mL of 10% FBS in PBS was added and the plates were incubated for 1 hr at room temperature. For the final washes, plates were first washed with PBS containing 0.05% Tween-20, followed by washing with PBS. Plates were developed by adding 20 μL of AEC (3-amino-9-ethyl-carbazole) chromogen per mL of AEC substrate (Sigma-Aldrich, Saint Louis, MO) and the reaction was stopped with water. After drying overnight, the plates were read on an AID ELISpot reader (Autoimmun Diagnostika GmbH, Strassberg, Germany). ELISpot results were determined by measuring the mean soft forming units (sfu) frequency of the antigen-stimulated sample minus the mean sfu frequency of the unstimulated sample (nil) and compared between the convalescent patients and subject controls. ROC curve analysis defined the overall positivity of S1 and S2 subunits responses by the IFN-γ ELISpot assays, and best cut-offs were determined to differentiate these study groups with the highest area under the curve (AUC). Subjects were considered to have a positive response when the mean number of IFN-γ sfu was greater than the determined best diagnostic cut-offs that for the specific antigen stimulation.

### Cytotoxicity Assay

We measured cytotoxic T-cell responses in HLA-A2-positive patients by xCELLigence^®^ system (Agilent, Santa Clara, CA). This system is a label free assay that can monitor cellular events in real time. The assay measures electrical impedance across micro-electrodes on the bottom of tissue culture E-Plates. The impedance measurement, expressed as Cellular Index (CI), provides quantitative information that can then give real time target lysis information.^40^ Human SKBR3 tumor cells, (which express MHC-I HLA-A2) were pulsed with the nine spike HLA-A2 peptides and seeded (5×10^3^ /well) into the wells of E-Plates in 100 μl of media. Cell adhesion and growth were monitored for up to 30 hrs until their exponential growth phase. Patient PBMCs (1×10^5^ per well) were added to the plates in a volume of 100 μl. Co-cultures were then assessed via electrical impedance every 5 mins for up to 60 hrs. Results, expressed as cellular index, were used in conjunction with the RTCA Software, and expressed as percentage lysis. = (CI SKBR3 only – (CI SKBR3 + T cells)) / (CI SKBR3 only) x100.

### Statistical Analysis

Results were compared using the Chi-square test for categorical variables (Fisher exact test for cells with numbers ≤ 5), as well as Pearson’s correlations and two-sided nonparametric Wilcoxon Rank-Sum test for continuous variables as appropriate. Cut-offs were determined for each antigenic conditions by ROC analysis to differentiate study groups of convalescents and unexposed individuals with the highest AUC. The percentage of T-cell phenotypes were reported as median and interquartile range. To present the data as per individual analysis, we followed bar graphs to demonstrate the proportion of SARS-CoV-2 anti-spike IgG ratios, CD4^+^ and CD8^+^ T cell phenotypes and IFN-γ ELISpot. To visually representation in the variations of T cell response, we generated heat maps with continuous color shading from each patient. In stimulation experiments, frequencies of activated T cells were adjusted by subtracting the unstimulated control value. P values ≤ 0.05 were considered statistically significant. Data were analyzed using JMP^™^ software, version 9.0.1 (SAS Institute, Inc., Cary, NC) and GraphPad Prism 9 (GraphPad Software, San Diego, CA).

## Results

**Figure 1:**
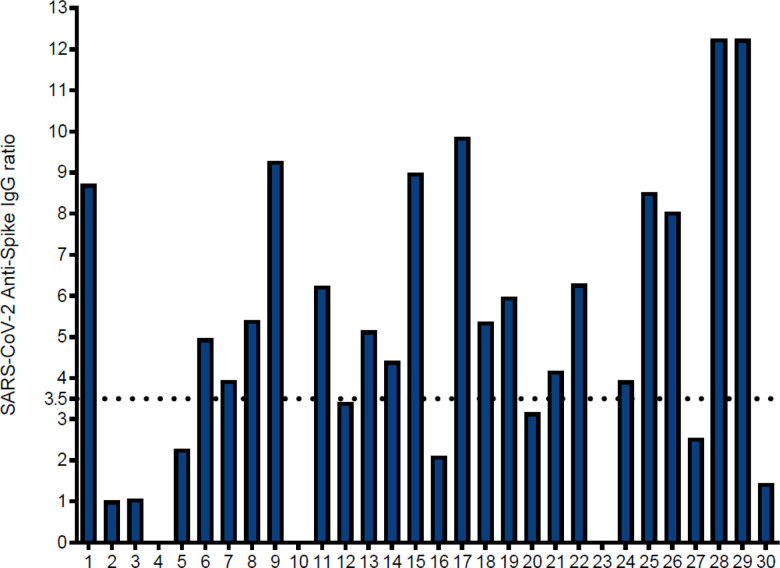
Anti-Spike IgG antibody response in convalescent subjects: Serum levels of IgG antibodies directed to S1 subunit were quantified by semiquantitative ELISA in convalescent subjects (n=30). Neutralizing antibody response was defined as an IgG ratio greater than or equal to 3.5 (horizontal dotted line). X axis shows the number of convalescent individuals with blank columns representing ≤zero response in the individual tested. Individuals 4, 10 and 23 did have a negative SARS-CoV-2 spike-specific antibody response, defined as a ratio of less than 0.8.

A total of 30 convalescent plasma donors and 7 unexposed controls were included in this study. The average age of the convalescent COVID-19 patients and the unexposed donors was 43 and 61 years, respectively (Table 2). Sixteen of 30 convalescent patients were female (53.3%), as were 4 out of 7 (57.1%) of the unexposed control individuals. Twelve of the convalescent patients were HLA-A2 positive (Table 2). A humoral anti-SARS-CoV-2 spike response was defined as an IgG ratio ≥ 3.5 (positive anti-spike neutralizing antibody response), corresponding to a neutralizing antibody titer ≥ 1:160.(20) Nineteen of the thirty (63.3%) convalescent patients had a positive neutralizing antibody response with the mean anti-SARS-CoV-2 IgG ratio being 5.03 (SD ± 3.49). However, in 11 convalescent patients (36.7%) IgG ratios were < 3.5, including three (10%) of the convalescent plasma donors having a negative anti-spike IgG measurement, defined as a ratio of less than 0.8 (Fig.1).

**Figure 2:**
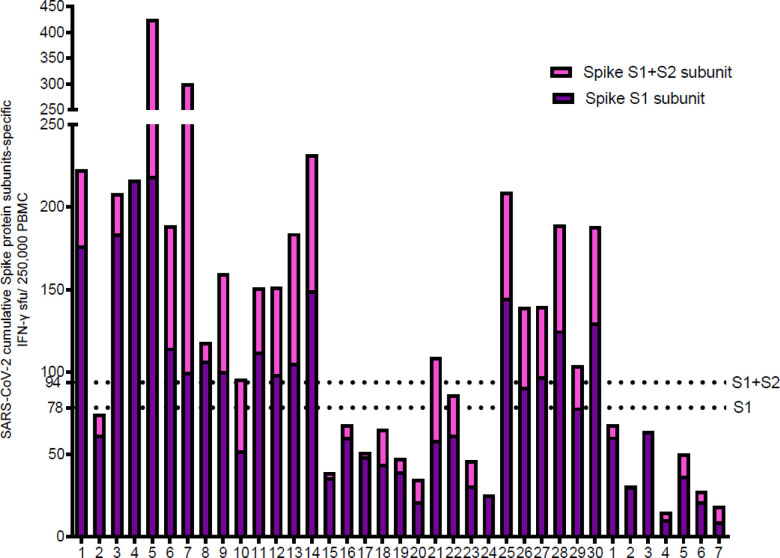
IFN-γ ELISpot assay to S1 and S2 subunits: Ex vivo IFN-γ ELISpot showing the magnitude and breadth of T cell responses in 30 convalescent and 7 unexposed subjects to S1 and S1+S2 subunits. The horizontal dotted lines represent the cutoffs of 78 and 94 sfu per 250,000 PBMCs with background subtracted based on the S1 subunit and S1 plus S2 subunit specific IFN-γ ELISpot responses, respectively to separate convalescent subjects from unexposed controls. Twenty-one of 30 convalescent patients (70%) and none of the unexposed donors showed a positive anti-spike IFN-γ ELISpot response by both S1 and S2 subunits.

**Figure 3:**
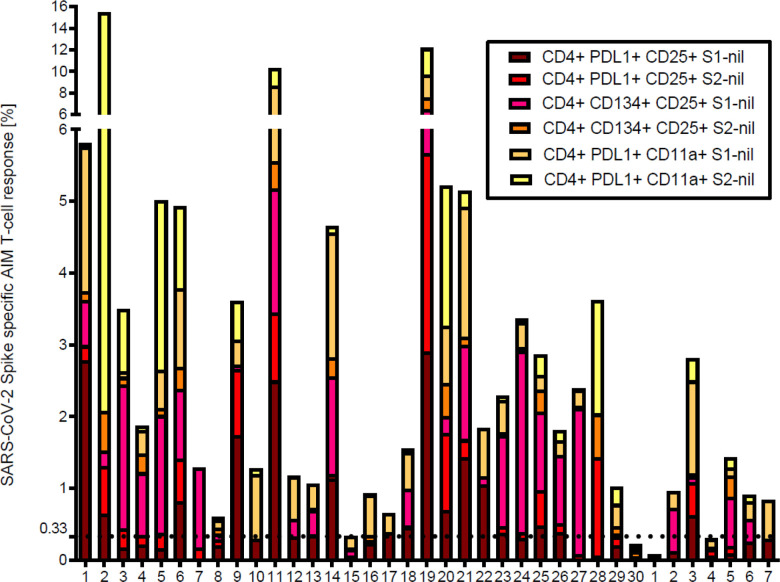
Cumulative FC AIM CD4^+^ T cell responses against S1 and S2 subunits: Flow cytometry-based characterization of S1 and S2 subunit specific CD4 T cell subsets (CD25^+^PDL1^+^, CD25^+^CD134^+^, PDL1^+^CD11a^+^) from convalescent patients and unexposed donors after 40 hrs *ex vivo* stimulated with S1 and S2 subunits. The displayed cumulative cut-off value of 0.33% (horizontal dotted horizontal line) was chosen as the cumulative lower limit of detection for the AIM CD4^+^ assays with the best diagnostic accuracy to differentiate convalescent vs. unexposed subjects for S1 and S2 subunits. All individual FC assay responses are background subtracted.

**Figure 4:**
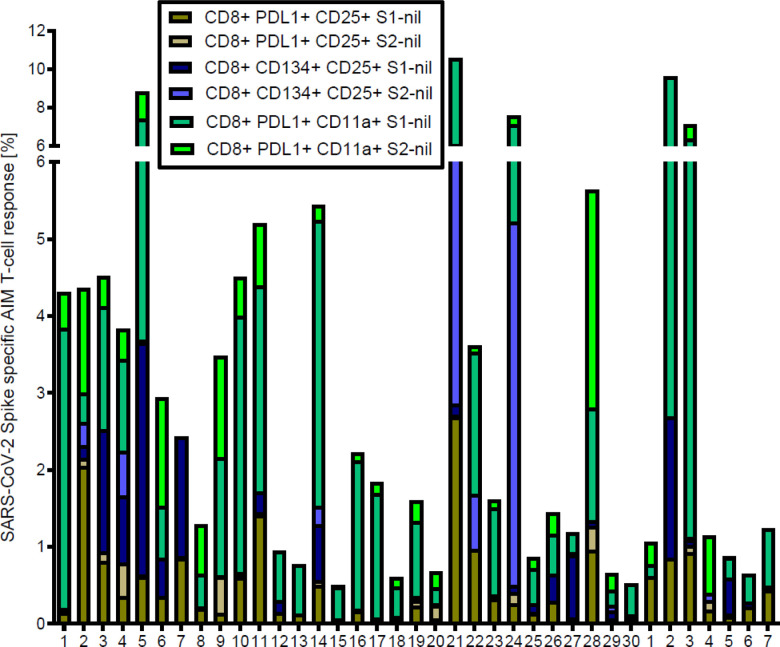
Cumulative FC AIM CD8^+^ T cell responses against S1 and S2 subunits: Flow cytometry-based characterization of S1 and S2 subunit specific CD8 T cell subsets (CD25^+^PDL1^+^, CD25^+^CD134^+^, PDL1^+^CD11a^+^) from convalescent patients and unexposed donors after 40 hrs ex vivo stimulated with S1 and S2 subunits. A cut-off value of 0.02% was chosen for the AIM CD8^+^ assays with the best diagnostic accuracy to differentiate convalescent vs. unexposed donors. No other subset had an area under the curve > 50%. All data plotted are background subtracted.

**Figure 5:**
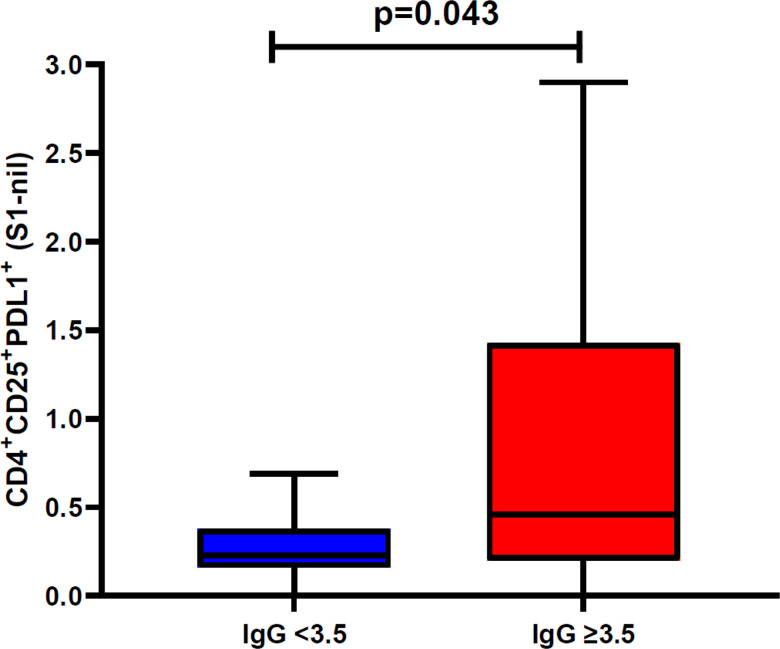
Association between Spike subunit 1 (S1) T cell response and anti IgG ratio ≥ 3.5: This box and whiskers plot represent the association between Spike subunit 1 (S1) speci c CD4^+^PDL1^+^CD25^+^ T-cell ≥ response and anti-SARS-CoV-2 IgG ratio with ≥ 3.5 (n=19) or <3.5 (n=10). None of the other T cell subsets were associated with SARS-CoV-2 spike-specific antibody response. Horizontal line represents median, and upper and lower boundaries of box represent 75th and 25th percentile. The whiskers extend from each quartile to the minimum and maximum. Statistical significance was calculated using the Wilcoxon Rank-Sum test.

The individual cellular antigen-specific responses against spike protein were measured by determining the cumulative IFN-γ ELISpot and AIM-FC assays for CD4 and CD8 results after ex vivo stimulation with the S1 and S2 subunits of SARS-CoV-2 (Fig. 2–5). Twenty-three of 30 convalescent donors (76.7%)had a positive anti-spike T-cell response based on the combined S1/S2 IFN-γ ELISpot response, which was determined by adding the best diagnostic antigen-specific IFN-γ response cut-offs that differentiate the groups of convalescent and unexposed individuals by ROC analysis for either IFN-γ ELISpot response to S1 subunit minus nil (cut off 78.3394 spots/2.5 × 10^5^ cells) and IFN-γ ELISpot response to S2 subunit minus nil (cut off ≥ 15.33 spots/2.5 × 10^5^ cells). None of the unexposed individuals were found to have positive IFN-γ ELISpot tests for the combined S1/S2 antigen response (Fig. 2).

We also evaluated the S1 and S2 specific individual AIM-FC CD4 and CD8 subsets to differentiate convalescent patients from unexposed donors. CD4 + CD25 + PD-L1+ (S2 subunit minus nil) had the highest AUC and reached statistical significance to differentiate the two study groups (P = 0.05) among all phenotypes. ROC analysis revealed that this subset showed 53.3% sensitivity and 85.7% specificity with the AUC of 0.7405 (**Supplementary Table: 1**). The cumulative AIM-FC antigen specific CD4^+^ and CD8^+^ lymphocytes against the S1 and S2 subunits of SARS-CoV-2 Spike protein responses for three surface marker subsets (CD25^+^PD-L1^+^, CD25^+^CD134^+^ and PD-L1^+^CD11a^+^) are shown in Figs. 3 & 4. Twenty-nine of the 30 convalescent patients, (96.7%) had a positive response by at least one of the AIM-FC subsets, including 6 out of 7 patients with negative IFN-γ ELISpot results. One patient was negative by both AIM-FC assays and by IFN-γ ELISpot; however, 4 out of the 7 unexposed subjects had measurable CD4^+^ or CD8^+^ T-cell response against the S1 and/or S2 subunits of SARS-CoV-2 spike protein by the AIM-FC assays, suggestive of cross-reactive immune response to other coronaviruses exposure(s) (Fig. 3, 4). Overall, 29 out of 30 patients (96.7%) had a measurable anti-Spike T-cell response with either IFN-γ ELISpot or AIM-FC assays and 23 out of 30 convalescent patients (76.7%) were positive for both types of tests. None of the SARS-CoV-2 antigen-specific activated CD4^+^ or CD8^+^ T cell subsets were associated with SARS-CoV-2 spike-specific antibody response. However, there was a statistically significant difference in the percentage of S1-specific CD4^+^CD25^+^PD-L1^+^ T cell response between convalescent patients with anti-SARS-CoV-2 S1 IgG ratio < 3.5 or ≥ 3.5, with a median percentage of 0.23% (IQR 0.16–0.38%) vs. 0.46% (IQR 0.20–1.43%), respectively (Wilcoxon Rank-Sum: P = 0.043) (Fig. 5).

**Figure 6:**
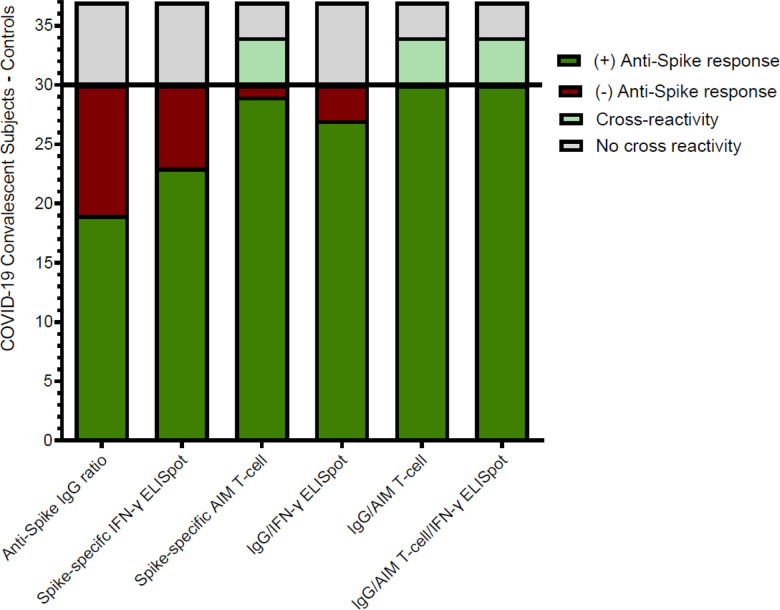
Comparison of humoral and cellular anti-Spike immune responses: This bar graph summarizes the overall positivity of anti-Spike IgG, IFN-γ ELISpot and AIM-FC data among convalescent patients (n=30) and unexposed donors (n=7). All convalescent patients had at least one positive result for humoral or cellular anti-spike immunity however AIM-FC also showed cross-reactivity/false positivity in 4 of 7 unexposed donors.

The individualized comparison of the anti-spike IgG ELISA, IFN-γ ELISpot, and AIM-FC Assays is shown in Fig. 6. Nineteen (63.3%) and 23 (76.7%) of 30 convalescent patients had a detectable humoral or cellular anti-spike T cell response by IgG ELISA and IFN-γ ELISpot, respectively. The combination of IgG ELISA and/or IFN-γ ELISpot was positive in 27 out of 30 patients (90%). None of the unexposed donors showed a cross reactive/false positive response for IgG ELISA or IFN-γ ELISpot. In contrast, anti-spike T-cell responses were detected by AIM-FC in 29 out of 30 convalescent patients and the remaining patient had a positive anti-spike IgG response (IgG ratio = 9.0). However, AIM-FC also showed substantial cross reactivity/false positivity in 4 out of 7 unexposed donors (57.1%). In summary, while all convalescent patients had at least one positive result for humoral or cellular anti-spike immunity using multimodality testing, there are concerns about the specificity of AIM-FC assay given the substantial amount of positivity in unexposed donors.

**Figure 7:**
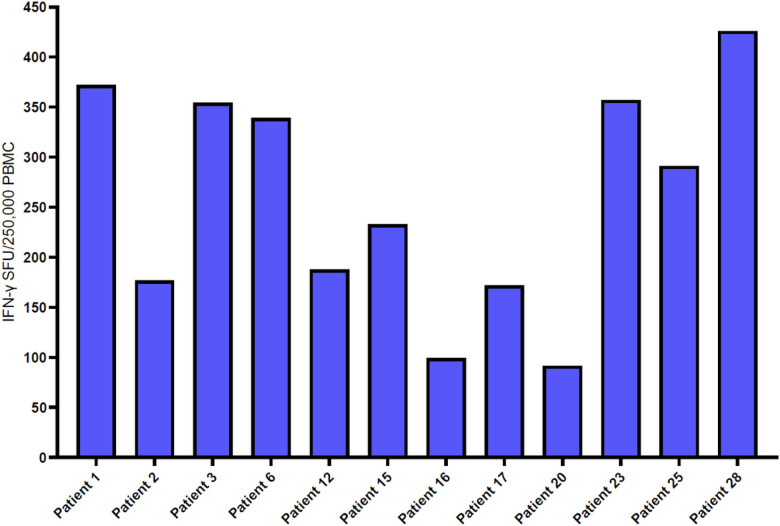
IFN-γ ELISpot responses against Spike peptides in the HLA-A2 positive cohort: This bar graph portrays cumulative Ex vivo IFN-γ ELISpot responses against the 9 HLA-A2 spike specific MHC-class I peptides from 12 HLA-A2 positive convalescent patients. All 12 patients had measurable IFN-γ ELISpot responses to nine HLA-A2 peptides but displayed a wide individual variety of responses. Responses are shown with the background subtracted.

The samples of 12 HLA-A2 positive convalescent patients were also tested by IFN-γ ELISpot, AIM-FC, and xCELLigence^®^ cytotoxicity assays against 9 HLA-A2 specific Spike MHC-I peptides. All 12 convalescent patients had measurable IFN-γ ELISpot response to tetanus toxoid (positive control) and the selected nine HLA-A2 peptides but showed wide variety (individual variability) of responses in the HLA-A2 positive subset of patients. (Fig. 7). We also evaluated the T-cell responses against nine spike HLA-A2 peptides in HLA-A2-positive patients by xCELLigence^®^ system. The cutoff for a positive test was ≥ 30% killing was fixed based on previous study.^41^ Ten of the 12 convalescent patients were determined to be positive based on SARS-CoV-2 spike specific cytotoxicity (Table 3). We observed that each patient had a unique response pro le to each of the peptides. For example, patient 12 had a response to 8 of the peptides, whereas patients 2 and 20 did not have a response to any of the peptides. The peptide Cov514 was unable to be recognized by any of the donors.

To visually representation in the variations of T cell immune response of 12 HLA-A2 positive convalescent patients, we generated heat maps with continuous color shading for each patient. The heatmaps demonstrating both CD4^+^ and CD8^+^ responses in relation to IFN-γ ELISpot and percentage of lysis by the cytotoxicity assay are seen in **Supplemental Fig. 1**. The convalescent patient 15 did not show measurable response by IFN-γ ELISpot and AIM-FC assays to S1 and S2 but positive IFN-γ ELISpot response to the HLA-A2 peptides.

## Discussion

Our data clearly demonstrates significant heterogeneity among anti-spike SARS-CoV2 adaptive immune responses of unvaccinated convalescent patients who successfully recovered from COVID-19 early during the pandemic. While no single immunoassay sufficiently identified all convalescent patients, comprehensive profiling of anti-spike adaptive immunity including ELISA, ELISpot, AIM-FC, and cellular cytotoxicity was able to identify a measurable adaptive anti-spike immune response in all subjects. These findings are in line with previous data demonstrating the value of comprehensive immune profiling to measure host immunity to various pathogens and vaccines. ^8,42–49^

A number of previous studies have examined adaptive immunity, both antibody and antigen specific T-cell responses to SARS-CoV-2 infection.^3,50,51^ These studies have largely focused on the characterization of the immune responses to different viral antigens including the spike protein and immunodominant peptide pools, and other membrane and nucleoproteins antigens using various measurement strategies such as measuring antibody response by various ELISA methods, Interferon gamma release assays by ELISpot, and FC assays as well as FC identification of antigen-specific T-cell activation based on different combinations of activation induced cell surface markers.^8^ Interestingly, while these studies clearly demonstrated that SARS-CoV-2 infection and COVID-19 vaccination induce both measurable humoral and cellular antigen specific immunity, the characteristics of a truly protective long-term anti-SARS-CoV-2 immune response remain unclear. Furthermore, besides a number of clinically implemented ELISA assays measuring anti-spike and anti-nucleocapsid antibodies against SARS-CoV-2, there has been a paucity of head-to-head comparison of clinically applicable approaches to measure anti-SARS-CoV-2 specific immunity, specifically T-cell responses. This discrepancy is probably largely due to significant heterogeneity in the quality and magnitude of measured adaptive anti SARS-CoV-2 immunity between patients within and between different methods utilized in these studies. Antigen-specific antibody responses have been reported to be more prevalent compared to T-cell responses, > 95% of convalescent patients have anti-SARS-CoV-2 antibodies if multiple ELISA assays are used.^21^ This may potentially be due to the timing of the testing in relationship to the disease onset. Furthermore, there is evidence that humoral immunity wanes over time while cellular immunity is more persistent.^52,53^ In our study we were able to detect T-cell response more frequently, however the detection of an anti-Spike T-cell response in almost all patients (96.7%) came at an expense of a substantial number of cross-reactive or false positive responses among unexposed individuals. Furthermore both antibody levels and T-cell response appear to vary based on age, gender, COVID-19 disease severity, the presence of preexisting immunity most likely related to prior exposures to other coronaviruses and other individual factors.^11,22,52,54–57^ Additionally, the level of humoral and cellular immunity varies based on the assays used to measure antibody (ELISA) and T-cell responses (ELISpot versus AIM-FC) and even based on different T-cell subsets analyzed by FC AIM assays. For example, CD4^+^ and CD8^+^ T-cell responses were only detectable in a minority of convalescent COVID-19 patients following severe infection when analyzed by FC using intracellular cytokine staining, but the opposite is true by utilizing AIM-FC methods with large pools of overlapping peptides.^16^ In addition, correlations between antibody and T-cell responses have been inconsistent in previously published data, and in our current study we also only observed an association between 1 of 12 CD4 and CD8 T cell subsets (CD4^+^PD-L1^+^CD25^+^ T-cell response to S1) evaluated by AIM-FC assays, stimulated with the S1 and S2 subunits of the spike protein. These and other observations indicate that humoral and adaptive antigen specific T-cell responses are probably regulated independently during SARS-CoV-2 infection^8,34,58^. In our study, contrast to previous studies, healthy unexposed donors commonly had a detectable lower T cell response against S2 by AIM-FC. ^31,59^ This might be due to the difference in the patient cohort as we recruited before the pandemic, the nature of the protein (as we used recombinant S2 subunit (S2,685–1211aa) rather than peptide pools that covers C-terminal portion (633–1273aa) in other studies), antigen stimulation period (40hrs versus 16hrs), type of analytes and other technical differences.

Interestingly, both antibody and cellular immune responses are essential for the clearance of the virus. This is seen in immunosuppressed patients with either HIV infection, hematological malignancies and therapeutic B-cell targeted immunosuppression who only develop partial immune responses resulting in chronic SARS-CoV-2 infection.^60,61^ This disease state is characterized by chronic low level viral replication and an inability to clear the virus due to defects in humoral and/or T-cell anti-SARS-CoV-2 responses.

A longitudinal study from Singapore also reported significant heterogeneity in the adaptive immune response among convalescent migrant workers infected during a COVID-19 outbreak early during the pandemic.^62^ While compared to our study these investigators did not use a similarly comprehensive approach to characterize the cellular immune responses, they also demonstrated significant heterogeneity during long-term follow up.^62^

Levels of neutralizing antibodies and T cells certainly represent important features of protective immunity. Specifically, our current data highlight that a comprehensive evaluation of anti-SARS-CoV-2 targeted immunity requires multiple immunological assessments using potentially multiple antigens and various immune assays measuring different aspects of the adaptive B and T cell responses. To our knowledge, our study represents one of the few datasets which include the evaluation of MHC-1 mediated cellular cytotoxicity in convalescent COVID-19 patients. The inclusion of the cellular cytotoxicity identified antigen specific cellular immune responses in most convalescent subjects (10 out of 12), including in one subject who had undetectable S1 and S2 responses by the IFN-γ ELISpot and AIM-FC assays, but a positive anti-SARS-CoV-2 spike antibody response.

Limitations of our study include the lack of information regarding patient comorbidities, long-term follow up and reinfection rates as well as the focus on the spike antigens of SARS-CoV2. The cohort is relatively small, and lack of patient COVID-19 severity information is another limitation as other studies demonstrated that this criteria would affect the subsequent immune response.^63,64^ Furthermore, while the fact that our convalescent patients were infected with SARS-CoV-2 early during the pandemic (April-May 2020) and before the introduction of COVID-19 vaccines provides a clean look at adaptive immune responses in the absence of vaccination or reinfection-induced confounding factors. However, the lack of exposure to more recent SARS-CoV2 variants and vaccination effect could limit the clinical applicability of our data to more recent times in the pandemic. Moreover, our study is limited by the small number of unexposed donors, however given the prevalence of COVID-19 infections and vaccination it would be almost impossible to recruit additional unexposed individuals, unless the samples were collected prior to the pandemic which comes with challenges for accurate immunoprofiling based on prolonged storage (> 3 years), especially for functional assays using old PBMC samples. In addition, some of our assays, specifically the assessment of cellular cytotoxicity is limited by the restriction of our approach to HLA-A2 positive individuals. This limitation could be potentially overcome by expanding this assessment by utilizing other MHC class I targeted peptide pools.

## Conclusion

In conclusion, our data clearly demonstrate that SARS-CoV-2 infection triggers significant humoral and cellular immunity in convalescent patients who successfully cleared the virus. However, in contrast to other infectious diseases adaptive immune responses against SARS-CoV2 infection appears to be very heterogenous. This heterogeneity may be attributable to individual viral load exposure, host factors, pre-existing cross-reactive immunity, COVID-19 disease severity, patient co-morbidities, and more recently, re-infections with SARS-CoV-2 variants and COVID-19 vaccinations. Furthermore, our data highlights the need for using multiple assays to comprehensively measure SARS-CoV-2 convalescent immune response to accurate identify correlates of cellular immunity. The observed heterogeneity of the immune response represents a very important consideration regarding the management of future COVID-19 pandemic waves and preventive and public health strategies, including measuring the immune response and effect of vaccinations to new SARS-CoV-2 variants and reinfections.

## Figures and Tables

**Table 1:** SARS-CoV-2 spike peptides predicted to bind HLA-A2 with a nity < 40 nM

N-terminal amino acid	Peptide sequence	Predicted affinity for HLA-A2
**268**	YLQPRTFLL	5.4
**132**	FQFCNDPFL	9.2
**690**	SIIAYTMSL	13.5
**385**	KLNDLCFTNV	15.3
**514**	FELLHAPATV	21
**3**	FLVLLPLV	28.2
**416**	KIADYNYKL	36.1
**1**	FVFLVLLPLV	32.6
**267**	GYLQPRTFLL	36.1

**Table 2:** Demographics of recruited study subjects

Demographic	Subjects, no. (%)		
	Convalescent patients (n = 30)	Controls (n = 7)	
Sex			P = 0.8110
Male	14 (46.7)	3 (57.1)	
Female	16 (53.3)	4 (42.9)	
Age (years)			P = 0.0397
Mean ± SD	44 ±15.4	61 ±16.8	
Range	21–67	35–80	
HLA-A2 +	12 (40)	N/A	

Nonparametric Wilcoxon Rank-Sum test was used for continuous variables. P values ≤ 0.05 were considered statistically significant.

**Table 3:** Cytotoxicity targeting the 9 HLA-A2 Peptides for SARS-CoV-2

SPIKE HLA-A2 peptides	Pt 1	Pt 2	Pt 3	Pt 6	Pt 12	Pt 15	Pt 16	Pt 17	Pt 20	Pt 23	Pt 25	Pt 28
**Cov1**	21.60	6.92	**43.89**	6.82	**47.53**	27.06	**46.33**	**61.67**	22.63	**35.27**	**30.85**	**58.08**
**Cov3**	**31.24**	8.13	**36.33**	21.94	**32.58**	**30.84**	27.34	**33.96**	19.92	**31.33**	**33.85**	**39.17**
**Cov132**	24.11	5.68	**31.66**	22.37	**31.45**	**33.23**	22.76	29.04	23.89	23.41	**36.42**	**54.43**
**Cov267**	25.93	3.21	26.52	25.95	**40.69**	**33.02**	27.05	**42.35**	20.17	**44.20**	**48.82**	**48.60**
**Cov268**	20.16	1.51	22.12	17.30	**42.98**	**31.81**	29.87	**37.64**	19.28	17.59	25.61	29.71
**Cov385**	5.48	8.82	21.48	17.10	**31.63**	22.94	21.91	21.10	5.57	11.01	18.32	24.79
**Cov416**	7.80	2.85	28.81	16.19	**36.67**	26.22	22.47	21.69	13.24	9.66	24.08	22.31
**Cov514**	10.30	4.94	24.79	13.79	10.79	13.92	14.88	14.80	12.96	22.89	5.10	18.34
**Cov690**	16.63	9.89	**35.59**	**31.67**	**39.34**	23.56	18.03	**62.20**	16.29	25.33	26.40	**38.35**
**# Peptides**	1.00	**0.00**	3.00	1.00	**8.00**	4.00	1.00	4.00	0.00	3.00	4.00	5.00

The values ≥ 30% cytotoxicity was indicated by Bold letters.

## Data Availability

The data that support the findings of this study are available from the corresponding author upon request.

## References

[1] Crotty S. Hybrid immunity. Science 372, 1392–1393, doi:10.1126/science.abj2258 (2021).

[2] DiPiazza A. T., Graham B. S. & Ruckwardt T. J. T cell immunity to SARS-CoV-2 following natural infection and vaccination. Biochemical and Biophysical Research Communications 538, 211–217, doi:10.1016/j.bbrc.2020.10.060 (2021). 33190827 PMC7584424

[3] Jarjour N. N., Masopust D. & Jameson S. C. T Cell Memory: Understanding COVID-19. Immunity 54, 14–18, doi:10.1016/j.immuni.2020.12.009 (2021). 33406391 PMC7749639

[4] Weiskopf D. Phenotype and kinetics of SARS-CoV-2-specific T cells in COVID-19 patients with acute respiratory distress syndrome. Science Immunology 5, 1–11, doi:10.1126/SCIIMMUNOL.ABD2071 (2020). PMC7319493 32591408

[5] Sekine T. Robust T Cell Immunity in Convalescent Individuals with Asymptomatic or Mild COVID-19. Cell 183, 158–168.e114, doi:10.1016/j.cell.2020.08.017 (2020). 32979941 PMC7427556

[6] Tarke A. Comprehensive analysis of T cell immunodominance and immunoprevalence of SARS-CoV-2 epitopes in COVID-19 cases. Cell Reports Medicine 2, 100204–100204, doi:10.1016/j.xcrm.2021.100204 (2021). 33521695 PMC7837622

[7] Rydyznski Moderbacher C. Antigen-Specific Adaptive Immunity to SARS-CoV-2 in Acute COVID-19 and Associations with Age and Disease Severity. Cell 183, 996–1012.1019, doi:10.1016/j.cell.2020.09.038 (2020). 33010815 PMC7494270

[8] Nguyen T. H. O. T Cells Targeting SARS-CoV-2: By Infection, Vaccination, and Against Future Variants. Frontiers in Medicine 8, doi:10.3389/fmed.2021.793102 (2021). PMC8739267 35004764

[9] Wiersinga W. J., Rhodes A., Cheng A. C., Peacock S. J. & Prescott H. C. Pathophysiology, Transmission, Diagnosis, and Treatment of Coronavirus Disease 2019 (COVID-19). JAMA 324, 782–782, doi:10.1001/jama.2020.12839 (2020). 32648899

[10] De Biasi S. Marked T cell activation, senescence, exhaustion and skewing towards TH17 in patients with COVID-19 pneumonia. Nature Communications 11, 3434–3434, doi:10.1038/s41467-020-17292-4 (2020). PMC7338513 32632085

[11] Brodin P. Immune determinants of COVID-19 disease presentation and severity. Nature Medicine 27, 28–33, doi:10.1038/s41591-020-01202-8 (2021). 33442016

[12] He R. The clinical course and its correlated immune status in COVID-19 pneumonia. Journal of Clinical Virology 127, 104361–104361, doi:10.1016/j.jcv.2020.104361 (2020). 32344320 PMC7152870

[13] Peng Y. Broad and strong memory CD4 + and CD8 + T cells induced by SARS-CoV-2 in UK convalescent individuals following COVID-19. Nature Immunology 21, 1336–1345, doi:10.1038/s41590-020-0782-6 (2020). 32887977 PMC7611020

[14] Björkander S. SARS-CoV-2 specific B- and T-cell immunity in a population-based study of young Swedish adults. Journal of Allergy and Clinical Immunology, doi:10.1016/j.jaci.2021.10.014 (2021). PMC8536496 34695490

[15] Boyton R. J. & Altmann D. M. The immunology of asymptomatic SARS-CoV-2 infection: what are the key questions? Nature reviews. Immunology, doi:10.1038/s41577-021-00631-x (2021). PMC8525456 34667307

[16] Olea B. Adaptive immune responses to SARS-CoV-2 in recovered severe COVID-19 patients. Journal of Clinical Virology 142, 104943–104943, doi:10.1016/j.jcv.2021.104943 (2021). 34391981 PMC8349475

[17] Bao Y. Dynamic anti-spike protein antibody profiles in COVID-19 patients. International Journal of Infectious Diseases 103, 540–548, doi:10.1016/j.ijid.2020.12.014 (2021). 33310028 PMC7836795

[18] Cox R. J. & Brokstad K. A. Not just antibodies: B cells and T cells mediate immunity to COVID-19. Nature Reviews Immunology 20, 581–582, doi:10.1038/s41577-020-00436-4 (2020). PMC7443809 32839569

[19] Long Q.-X. Clinical and immunological assessment of asymptomatic SARS-CoV-2 infections. Nature Medicine 26, 1200–1204, doi:10.1038/s41591-020-0965-6 (2020). 32555424

[20] Okba N. M. A. Severe Acute Respiratory Syndrome Coronavirus 2-Specific Antibody Responses in Coronavirus Disease Patients. Emerging Infectious Diseases 26, 1478–1488, doi:10.3201/eid2607.200841 (2020). 32267220 PMC7323511

[21] Dan J. M. Immunological memory to SARS-CoV-2 assessed for up to 8 months after infection. Science 371, eabf4063–eabf4063, doi:10.1126/science.abf4063 (2021). 33408181 PMC7919858

[22] Havervall S. Robust humoral and cellular immune responses and low risk for reinfection at least eight months following asymptomatic to mild COVID-19. Journal of Internal Medicine, joim.13387-joim.13387, doi:10.1111/joim.13387 (2021). PMC8661920 34459525

[23] Gallais F. Evolution of antibody responses up to 13 months after SARS-CoV-2 infection and risk of reinfection. EBioMedicine 71, 103561–103561, doi:10.1016/j.ebiom.2021.103561 (2021). 34455390 PMC8390300

[24] Liu L. Striking Antibody Evasion Manifested by the Omicron Variant of SARS-CoV-2. Nature, doi:10.1038/s41586-021-04388-0 (2021). 35016198

[25] Carreño J. M. Activity of convalescent and vaccine serum against SARS-CoV-2 Omicron. Nature, doi:10.1038/s41586-022-04399-5 (2021). 35016197

[26] Planas D. Considerable escape of SARS-CoV-2 Omicron to antibody neutralization. Nature, doi:10.1038/s41586-021-04389-z (2021). 35016199

[27] Thorne L. G. Evolution of enhanced innate immune evasion by SARS-CoV-2. Nature, doi:10.1038/s41586-021-04352-y (2021). PMC8850198 34942634

[28] Cele S. Omicron extensively but incompletely escapes P zer BNT162b2 neutralization. Nature, doi:10.1038/s41586-021-04387-1 (2021). PMC8866126 35016196

[29] Cao Y. Omicron escapes the majority of existing SARS-CoV-2 neutralizing antibodies. Nature, doi:10.1038/s41586-021-04385-3 (2021). PMC8866119 35016194

[30] Ni L. Detection of SARS-CoV-2-Specific Humoral and Cellular Immunity in COVID-19 Convalescent Individuals. Immunity 52, 971–977.e973, doi:10.1016/j.immuni.2020.04.023 (2020). 32413330 PMC7196424

[31] Grifoni A. Targets of T Cell Responses to SARS-CoV-2 Coronavirus in Humans with COVID-19 Disease and Unexposed Individuals. Cell 181, 1489–1501.e1415, doi:10.1016/j.cell.2020.05.015 (2020). 32473127 PMC7237901

[32] Le Bert N. SARS-CoV-2-specific T cell immunity in cases of COVID-19 and SARS, and uninfected controls. Nature 584, 457–462, doi:10.1038/s41586-020-2550-z (2020). 32668444

[33] Mateus J. Selective and cross-reactive SARS-CoV-2 T cell epitopes in unexposed humans. Science 370, 89–94, doi:10.1126/science.abd3871 (2020). 32753554 PMC7574914

[34] Tan A. T. Early induction of functional SARS-CoV-2-specific T cells associates with rapid viral clearance and mild disease in COVID-19 patients. Cell Reports 34, 108728–108728, doi:10.1016/j.celrep.2021.108728 (2021). 33516277 PMC7826084

[35] Sahin U. BNT162b2 vaccine induces neutralizing antibodies and poly-specific T cells in humans. Nature 595, 572–577, doi:10.1038/s41586-021-03653-6 (2021). 34044428

[36] Folegatti P. M. Safety and immunogenicity of the ChAdOx1 nCoV-19 vaccine against SARS-CoV-2: a preliminary report of a phase 1/2, single-blind, randomised controlled trial. The Lancet 396, 467–478, doi:10.1016/S0140-6736(20)31604-4 (2020). PMC7445431 32702298

[37] Andreatta M. & Nielsen M. Gapped sequence alignment using artificial neural networks: application to the MHC class I system. Bioinformatics (Oxford, England) 32, 511–517, doi:10.1093/bioinformatics/btv639 (2016). 26515819 PMC6402319

[38] Escalante P. Combinatorial immunopro ling in latent tuberculosis infection: Toward better risk strati cation. American Journal of Respiratory and Critical Care Medicine 192, 605–617, doi:10.1164/rccm.201412-2141OC (2015). 26030344 PMC4595688

[39] Bowyer G. Activation-induce markers detect vaccine-specific CD4 + T cell responses not measured by assays conventionally used in clinical trials. Vaccines 6, doi:10.3390/vaccines6030050 (2018). PMC6161310 30065162

[40] Agilent. xCELLigence RTCA S16 - Pilot Scale RUO, <https://www.agilent.com/en/product/cell-analysis/real-time-cell-analysis/rtca-analyzers/xcelligence-rtca-s16-pilot-scale-741231> (2022)

[41] Azqueta A., Stopper H., Zegura B., Dusinska M. & Møller P. Do cytotoxicity and cell death cause false positive results in the in vitro comet assay? Mutat Res Genet Toxicol Environ Mutagen 881, 503520, doi:10.1016/j.mrgentox.2022.503520 (2022). 36031332

[42] Sridhar S. Longevity and Determinants of Protective Humoral Immunity after Pandemic In uenza Infection. American Journal of Respiratory and Critical Care Medicine 191, 325–332, doi:10.1164/rccm.201410-1798OC (2015). 25506631 PMC4351579

[43] Mantei A. Mycobacterium tuberculosis -specific CD4 T-cell scoring discriminates tuberculosis infection from disease. European Respiratory Journal, 2101780–2101780, doi:10.1183/13993003.01780-2021 (2022). 35618277 PMC9329623

[44] Niessl J., Sekine T. & Buggert M. T cell immunity to SARS-CoV-2. Seminars in Immunology, 101505–101505, doi:10.1016/j.smim.2021.101505 (2021). 34711489 PMC8529278

[45] Silva L. T. d. SARS-CoV-2 recombinant proteins stimulate distinct cellular and humoral immune response pro les in samples from COVID-19 convalescent patients. Clinics 76, doi:10.6061/clinics/2021/e3548 (2021). PMC8610223 34878034

[46] Valletta J. J. & Recker M. Identification of immune signatures predictive of clinical protection from malaria. PLOS Computational Biology 13, e1005812–e1005812, doi:10.1371/journal.pcbi.1005812 (2017). 29065113 PMC5669498

[47] Dan J. M. A Cytokine-Independent Approach To Identify Antigen-Specific Human Germinal Center T Follicular Helper Cells and Rare Antigen-Specific CD4 + T Cells in Blood. The Journal of Immunology 197, 983–993, doi:10.4049/jimmunol.1600318 (2016). 27342848 PMC4955771

[48] Dijkman K. Pulmonary MTBVAC vaccination induces immune signatures previously correlated with prevention of tuberculosis infection. Cell Reports Medicine 2, 100187–100187, doi:10.1016/j.xcrm.2020.100187 (2021). 33521701 PMC7817873

[49] Lewinsohn D. M. & Lewinsohn D. A. The Missing Link in Correlates of Protective Tuberculosis Immunity: Recognizing the Infected Cell. Frontiers in Immunology 13, doi:10.3389/mmu.2022.869057 (2022). PMC9040373 35493495

[50] Swadling L. & Maini M. K. T cells in COVID-19 — united in diversity. Nature Immunology 21, 1307–1308, doi:10.1038/s41590-020-0798-y (2020). 32895541

[51] Boonyaratanakornkit J. Clinical, laboratory, and temporal predictors of neutralizing antibodies against SARSCoV-2 among COVID-19 convalescent plasma donor candidates. The Journal of clinical investigation 131, doi:10.1172/JCI144930 (2021). PMC7843229 33320842

[52] Lu Z. Durability of SARS-CoV-2-speci c T cell responses at 12-months post-infection. The Journal of Infectious Diseases, doi:10.1093/infdis/jiab543 (2021). PMC8672777 34673956

[53] Feng C. Protective humoral and cellular immune responses to SARS-CoV-2 persist up to 1 year after recovery. Nat Commun 12, 4984, doi:10.1038/s41467-021-25312-0 (2021). 34404803 PMC8370972

[54] Brunk F. SARS-CoV ‐2 ‐reactive T ‐cell receptors isolated from convalescent COVID 19 patients confer potent T cell effector function. European Journal of Immunology 51, 2651–2664, doi:10.1002/eji.202149290‐ (2021). ‐ 34424997 PMC8646365

[55] Almendro-Vázquez P. Longitudinal dynamics of SARS-CoV-2-specific cellular and humoral immunity after natural infection or BNT162b2 vaccination. PLoS pathogens 17, e1010211–e1010211, doi:10.1371/journal.ppat.1010211 (2021). 34962970 PMC8757952

[56] Jacot D. Evaluation of sixteen ELISA SARS-CoV-2 serological tests. Journal of Clinical Virology 142, 104931–104931, doi:10.1016/j.jcv.2021.104931 (2021). 34365228 PMC8295192

[57] Zhou J. Viral emissions into the air and environment after SARS-CoV-2 human challenge: a phase 1, open label, rst-in-human study. Lancet Microbe, doi:10.1016/S2666-5247(23)00101-5 (2023). PMC10256269 37307844

[58] Elyanow R. T cell receptor sequencing identifies prior SARS-CoV-2 infection and correlates with neutralizing antibodies and disease severity. JCI insight 7, doi:10.1172/jci.insight.150070 (2022). PMC9220924 35439166

[59] Braun J. SARS-CoV-2-reactive T cells in healthy donors and patients with COVID-19. Nature 587, 270–274, doi:10.1038/s41586-020-2598-9 (2020). 32726801

[60] Marty P. K. Antigen Specific Humoral and Cellular Immunity Following SARS-CoV-2 Vaccination in ANCA-Associated Vasculitis Patients Receiving B-Cell Depleting Therapy. Frontiers in immunology 13, 834981–834981, doi:10.3389/mmu.2022.834981 (2022). 35154159 PMC8831839

[61] Hueso T. Convalescent plasma therapy for B-cell-depleted patients with protracted COVID-19. Blood 136, 2290–2295, doi:10.1182/blood.2020008423 (2020). 32959052 PMC7702482

[62] Le Bert N. Widely heterogeneous humoral and cellular immunity after mild SARS-CoV-2 infection in a homogeneous population of healthy young men. Emerging Microbes & Infections 10, 2141–2150, doi:10.1080/22221751.2021.1999777 (2021). 34709140 PMC8604544

[63] Mukund K. Immune Response in Severe and Non-Severe Coronavirus Disease 2019 (COVID-19) Infection: A Mechanistic Landscape. Front Immunol 12, 738073, doi:10.3389/mmu.2021.738073 (2021). 34721400 PMC8548832

[64] García-González P. Dysregulated Immune Responses in COVID-19 Patients Correlating With Disease Severity and Invasive Oxygen Requirements. Front Immunol 12, 769059, doi:10.3389/mmu.2021.769059 (2021). 34745145 PMC8567168

